# Teachers’ responses to racism and racist bullying in Dutch primary schools

**DOI:** 10.3389/fpsyg.2024.1393719

**Published:** 2025-01-23

**Authors:** Karen M. A. Sieben-Aduful, Roy A. Willems, Trijntje Völlink, Nico van der Wiel, Maria Sapouna, Pieter de Bruijn, Gemma Blok, Arjan E. R. Bos, Giel van Lankveld

**Affiliations:** ^1^Faculty of Psychology, Open Universiteit, Heerlen, Netherlands; ^2^School of Education and Social Sciences, University of the West of Scotland, Paisley, United Kingdom; ^3^Faculty of Humanities, Open Universiteit, Heerlen, Netherlands; ^4^Faculty of Humanities, University of Utrecht, Utrecht, Netherlands; ^5^Centre for Public Health, Healthcare and Society, National Institute for Public Health and the Environment (RIVM), Bilthoven, Netherlands; ^6^Faculty of Educational Sciences, Open Universiteit, Heerlen, Netherlands

**Keywords:** racism, racist bullying, perceived competence, class climate, educational material, support needs, teacher responses

## Abstract

**Background:**

Early in life, children with a non-White skin color, or a non-Western cultural or religious background, are susceptible of experiencing acts of racism. Since they spend a significant time of their daily life in school, teachers have a crucial role in providing a safe and bias-free environment for these children. However, teachers might find it challenging to react to bullying based on racism.

**Aim:**

This study aims to investigate teachers’ experiences of and reactions to racist bullying among pupils in primary schools in The Netherlands. Additionally, this study aims to explore how racism is discussed in class.

**Method:**

With nine semi-structured in-depth interviews teachers’ attitudes and reactions toward racist bullying were examined as well as their perceived competence and skills of handling this. In addition, teachers’ availability on tools to discuss racism in class was investigated as well. Data was analyzed using thematic analysis.

**Results:**

The results show that teachers emphasize a safe class climate but find it challenging addressing racist incidents, for instance determining its severity, or coping with the external influences on pupils’ racist beliefs. Most teachers also expressed to not make a distinction between racist and interpersonal bullying. While teachers expressed that they talked about racism in the classroom, it seemed that they primarily talked about cultural diversity and not racism per se.

**Conclusion:**

Dealing with racism and racist bullying is an important but complicated task for teachers. Providing teachers with appropriate tools to increase knowledge, awareness and skills will help them to understand the negative impact racism has on children. Further, the results implicate that a pro-active approach can stimulate teachers to critically reflect on their own racial identity, and on school methods, teaching resources and policies concerning racism.

## Introduction

Racist bullying is a serious problem. It is the second most frequent reason for bullying victimization among pupils, after bullying victimization due to appearance (United Nations Educational, Scientific and Cultural Organization (UNESCO), 2019). Approximately one in seven of all bullied children are bullied because of their race, nationality, skin color or religion (United Nations Educational, Scientific and Cultural Organization (UNESCO), 2019). Sapouna et al. (2022) indicate in their systematic literature review that racist bullying victimization is a problem in various countries. Racist bullying is a form of biased-based bullying. Biased-based bullying can be defined as bullying in an intergroup context whereby victims belong to groups that have historically stigmatized individual characteristics or identities (Eisenberg et al., 2022; Palmer and Abbott, 2018). Racist bullying can be defined as a specific interpersonal form of bullying based on one’s skin color, ethnicity, cultural, or religious background (Goldberg, 2006). It is influenced by racist norms and values that circulate in societies, which are then expressed in schools (Jones et al., 2023). Racism can be defined as the perpetuation or exacerbation of unequal opportunities among ethnoracial groups (Berman and Paradies, 2010) and should be understood as a structural and societal problem (Berman and Paradies, 2010; Bonilla-Silva, 1997; Sijpenhof, 2020). Teachers are responsible for meeting children’s safety needs by creating a safe social and inclusive environment since children spend considerable time in school. Teachers also have an important role in recognizing and responding to racist bullying and preventing it in order to maintain this safe environment. To tackle racist bullying effectively, teachers need to be able to address race and racism in class. This, however, is an important challenge for teachers (Boutte et al., 2011; Castagno, 2008; Jones et al., 2023; Perez, 2022; Yared et al., 2020).

### Excluding race and racism from the discourse within schools

Various studies demonstrate how race and racism are discussed within schools. Race and racism are often not part of the discourse within schools (Castagno, 2008), and accordingly, teachers are often reluctant to discuss these sensitive topics (Vass, 2013). The reason for this unwillingness has been attributed to feelings of discomfort (Boutte et al., 2011; Henze et al., 1998), being unprepared (Perez, 2022; Tropp and Rucinski, 2022), or not being confident or competent enough (Yared et al., 2020). When racism does become a topic in the classroom, it is often downsized and minimized (Jones et al., 2023) or silenced to some extent (Henze et al., 1998; Rosvall and Öhrn, 2014; Yared et al., 2020). This situation is perhaps unsurprising since there is a strong desire for comfort and ideological safety within classrooms. Avoiding discussion about race and racism, consequently, creates an illusion of issues of race not being important or that they do not exist (Castagno, 2008). Children can thus develop a false belief of racism not being a problem in society (Husband, 2012). Excluding race and racism from the discourse stimulates a reinforcement and perpetuation of stereotypical and racist beliefs among pupils (Castagno, 2008).

### Racist bullying in Dutch primary schools

Over the past decades, Dutch society has become increasingly culturally diverse, which to a large extent stems from a significant increase in first and second generation migrants from the former colonies of Suriname and the Dutch Antilles, as well as from Turkey and Morrocco with whom the Netherlands had signed migrant worker agreements that ran from 1964 until 1973 (CBS Statline, 2022). Postcolonial migration from Suriname grew strongly in the 1970s, with the country gaining independence in 1975. With the Dutch Antilles formally remaining part of the Kingdom of the Netherlands, migration from this region shows a more recent growth (Oostindie, 2012). When people from these and other former slave colonies started migrating to The Netherlands racist beliefs toward these migrants were externalized (Rose, 2022). During colonial times, the Netherlands was heavily involved in the transatlantic slave trade, the history of which, and particularly its gruesome aspects, for a long time were largely ignored in Dutch society. For example, the period from the late sixteenth to the late seventeenth century has often been described as the ‘Dutch Golden Age’, focusing on the commercial successes of the Dutch East India Company (VOC), whilst neglecting the company’s aggressive military strategies and involvement in slavery, as well as the history of the West Indies Company (WIC), which played a major role in the transatlantic slave trade in that same period (Klein, 2017). However, awareness of Dutch colonial history has increased over the last couple of years, with, for instance, the government offering formal apologies in 2022 for the country’s role in the history of the slave trade (Allen et al., 2023). The history of colonialism, and its legacy of discrimination and racism, is still a sensitive subject in Dutch society, which also manifests itself through controversy around heritage sites and practices, such as statues and traditions, that can be linked to colonial times (e.g., Rodenberg and Wagenaar, 2016).

Even today racism is still a serious problem in The Netherlands. For instance, people with a Muslim background experience more racism in The Netherlands on average in comparison to other European countries (European Union Agency for Fundamental Rights, 2024). Even though the previous mentioned study of the European Union has been conducted among adults, it can be expected that children experience racism as well. Verkuyten and Thijs (2002) indicate that 38% of pupils with a Turkish, Moroccan or Suriname background experience racist bullying in Dutch primary schools during their schooling period, whereby pupils’ expectations of the teacher’s response influence whether or not racist bullying is reported. This indicates that teachers play an important role in racist bullying experiences of pupils. Even though recent data on racist bullying in Dutch primary schools seems to be limited, the study of Andriessen et al. (2020) demonstrates that more than 50% of Dutch students with a Turkish, Moroccan, Surinam, or Antillean background, among various school levels, experienced racism within a school year. This shows that racism still is a relevant problem. Racist bullying can not only be explicit, such as name calling or ridiculing skin color, ethnicity, or religion, but it can also be implicit, hidden in other indirect and aversive forms of bullying, such as avoidance and social exclusion (Sullivan, 2005). Discussing racism in classrooms may be a challenge, yet teachers need to recognize that children do experience racism (Boutte et al., 2011), and very likely racist bullying as well.

### Personal and environmental factors influencing how teachers handle racism and racist bullying

Several personal and environmental factors seem to play an important role in how teachers handle racism and racist bullying in the classroom. Personal factors include specific beliefs and attitudes about, for instance race (Alvarez and Milner 2018; Hinojosa and Moras, 2009; Soto, 2022) and citizenship (De Schaepmeester et al., 2022). Personal factors also include knowledge about racism and skills in addressing racism or tackling racist bullying (Hyseni Duraku et al., 2022; Perez, 2022), competence in discussing racism and tackling racist bullying (De Luca et al., 2019; Perez, 2022; Wing Sue et al., 2009; Yared et al., 2020), and the understanding of one’s own racial identity (Dulin-Keita et al., 2011; Johnson, 2002; Perez, 2022; Tatum, 1992; Tettegah, 1996). Environmental factors that play a role in teachers’ approaches and strategies toward handling race and racism are, for instance, the classroom climate (De Schaepmeester et al., 2022; Henze et al., 1998; Longobardi et al., 2018), the school climate (Cohen et al., 2009; Desombre et al., 2021; Perez, 2022; Walton et al., 2014; Webster et al., 2010), and school curriculum (Ali, 2018; Wills, 2019). How these personal and environmental factors might influence teachers’ responses to racist bullying and discussing racism will be explained in the following section.

#### Personal factors

##### Beliefs and attitudes

Beliefs and attitudes are important personal factors that affect teachers’ approaches toward racism and racist bullying (Fishman et al., 2021; Smithies, n.d.) Teachers’ beliefs and attitudes about, for example, citizenship and cultural diversity, impact their approaches and practices, including what strategies they use to reduce racism (De Schaepmeester et al., 2022). Teachers’ beliefs and attitude toward the cultural background of the pupils can also determine to what extent racism is discussed in class. Teachers teaching classes with a larger number of pupils of diverse cultural backgrounds tend to discuss racism more often than teachers who have a higher proportion of White pupils (Walton et al., 2014). Furthermore, teachers’ beliefs and attitudes toward race and certain cultural groups can also affect how they approach pupils from various cultural backgrounds (Hinojosa and Moras, 2009). Their beliefs and attitudes can particularly reinforce the negative expectations teachers already have of these pupils, which consequently impacts the school performances and educational experiences of these pupils (Morris, 2022; Soto, 2022). Alvarez and Milner (2018) show that even if teachers’ beliefs about the importance of discussing race and racism are present, the feeling of unpreparedness, due to fear and discomfort, results in teachers not discussing these topics in class. The negative emotion of fear, in this case, is stronger than the positive belief and hence impacts the attitude and behavior of these teachers. This feeling of unpreparedness can be tackled by a feeling of competence through the right knowledge, skills and tools (Perez, 2022).

##### Knowledge and skills

Knowledge and skills are also important personal factors that can influence teachers’ approaches toward racism and racist bullying as well (Winterton et al., 2006). Teachers require knowledge about racist bullying and skills to intervene in racist bullying. Lacking knowledge and skills about how to intervene in a racist incident is, in fact, an obstacle for teachers (Wing Sue et al., 2009). Another important aspect is the need for professional development to increase teachers’ knowledge about racism, and to acquire better skills to teach about it (Acosta and Ackerman-Barger, 2017). In particular, teachers need professional development to develop knowledge and skills on how to deal with the emotional aspects of discussing race and racism, if not, it can become very challenging to teach about racism (Perez, 2022). Even though professional development contributes to increasing knowledge and skills on teaching about racism, after completion teachers might still find it challenging to begin discussions about race and racism in the classroom (Smith et al., 2017). According to Perez (2022), teachers must approach learning about race and racism as an ongoing intentional journey instead of a place of arrival. Professional development about race and racism should therefore not just be an occasion but rather a long-term goal. It will then enhance teachers’ knowledge, skills and competencies for teaching strategies and improve the quality of education continuously (Hyseni Duraku et al., 2022).

##### Competence

Another personal factor that influences teachers’ approaches toward discussing racism and addressing racist bullying is their perceived competence (Ryan and Deci, 2020). Even though teachers might have certain beliefs and attitudes about the importance of both discussing racism and tackling racist bullying, they can still feel incompetent in doing so. Competence depends on knowledge and skills (Winterton et al., 2006). For example, a low perceived competence, due to the fear of losing control over a classroom when discussing race and racism, is a barrier that hinders teachers from discussing racism in class (Wing Sue et al., 2009). It is crucial that teachers feel competent enough to engage with pupils from different cultural backgrounds to discuss racism and the experiences of their pupils (Yared et al., 2020). Besides a low perceived competence to discuss race and racism in class, many teachers also feel incompetent in recognizing racist incidents and perceive this as a hindrance as well (Wing Sue et al., 2009). De Luca et al. (2019) have shown that teachers who feel incompetent are more passive and observant, whereas teachers who feel competent and intervene during racist incidents can have a positive effect on pupils. When teachers intervene, pupils tend to report bullying incidents more often than with teachers who have a passive attitude, this consequently affects the extent and level of racist bullying in school (De Luca et al., 2019). Therefore, if teachers are more equipped with the right knowledge, skills and tools to teach about race and racism and to attend to racist incidents, this will not only increase their own perceived competence, but it is also expected that they will be perceived as more competent by others (Wing Sue et al., 2009). Teachers feeling competent enough in addressing racist incidents is essential in order for them to attend, interpret and respond to racist incidents (Shah and Coles, 2020).

#### Environmental factors

##### Classroom climate

An environmental factor that influences teachers’ approaches toward racism is the classroom climate. A positive classroom climate depends on a range of factors, such as healthy teacher-pupil relationships, the opportunity to debate and discuss, taking children’s voices seriously, meaningful learning, and whether there is space created to discuss controversial issues and moral dilemmas (De Schaepmeester et al., 2022). More specifically, the teacher-pupil relationships can affect intergroup relations of pupils and attitudes toward racist bullying (Longobardi et al., 2018). In addition, if teachers decide to discuss racism in class, then it needs to be done in a safe environment, and children need to feel safe enough to reveal personal information and opinions (Henze et al., 1998).

##### School climate

Another environmental factor that influences teachers’ approaches toward racist bullying is the school climate (Cohen et al., 2009). The school climate is shaped by the social environment of the teacher, which consists of the school director, colleagues, and parents. How the school handles race and racism can influence teachers’ approaches toward racist bullying (Walton et al., 2014). On the one hand, supportive behavior in the school impacts the attitude and behavior of teachers positively (Desombre et al., 2021). On the other hand, a lack of institutional support (Perez, 2022), good leadership (Webster et al., 2010), commitment to challenge racism in schools or a lack of understanding from colleagues (Pearce, 2014), can hinder teachers from discussing race and racism in class. This hindrance even occurs if teachers hold positive beliefs and attitudes toward the importance of addressing race and racism in class (Desombre et al., 2021).

##### School curriculum

A final environmental factor that influences teachers’ approaches toward racism is the educational material. Educational material that supports discussions about racism can help teachers navigate this challenging and emotional discourse. The educational material can be used as a conversation starter and give teachers confidence to discuss racism (Wills, 2019). To have educational material on race and racism, it is essential that these topics are already integrated into both the curriculum and syllabus. Including race and racism in the curriculum can help in learning about different cultural perspectives, create awareness and engaging dialogues to deconstruct damaging beliefs, and empower both teachers and pupils (Ali, 2018). If the educational material ignores racism, then that is another way of silencing and ignoring its actual existence. However, if educational material does include information about race and racism, it is of importance that the material provides correct and complete information and that teachers understand what they are teaching (Wills, 2019).

### This study

Teachers need to be better prepared to contribute productively to discussing race and racism in class (Vass, 2013). As previous studies indicate, race and racism are often excluded from the discourse within schools. In the Netherlands, this is the case as well. Teachers likewise feel uncomfortable discussing race and racism (Sijpenhof, 2020) and silence discussions about racism (Sijpenhof, 2021). They not only find it uncomfortable but also challenging to connect historical aspects of racism with contemporary forms of inequality (Sijpenhof, 2021). This study aims to investigate how the previously mentioned personal and environmental factors play a role in how teachers of Dutch primary schools approach racist bullying and discuss racism in class. A qualitative approach is used to explore the teachers’ understandings of these factors, for example by analyzing how their responses to racist bullying and approaches toward discussing racism are formed by their attitudes and their underlying beliefs about racism. With this understanding this study can provide the necessary knowledge to help develop materials and tools for the specific needs of teachers in effectively tackling racist bullying and discussing racism in class. It is suggested that teachers who confidently discuss racism in class may be better equipped to address racist bullying in school.

This study addresses the following research questions:

- What are teachers’ attitudes and beliefs about racist bullying among pupils and about teaching racism in Dutch primary schools?
- What are teachers’ experiences of and reactions toward racist bullying among pupils?
- What are the necessary skills and tools to approach racist bullying among pupils, and to teach about racism?
- What is the perceived competence of teachers on how to deal with racism?

Within the scope of this study, face-to-face semi-structured in-depth interviews were chosen as an exploratory qualitative method to gain insight into teachers’ experiences and perspectives of racism and racist bullying in primary school.

## Methods

### Ethical approval

This study is approved by the Research Ethics Committee of the Open Universiteit of the Netherlands, with reference number RP108/U202302981.

### Sampling and recruitment

Inclusion criteria were teachers who had working experiences in the 7th or 8th year of Dutch mixed primary schools. Grades 7 and 8 are the last 2 years of Dutch primary schools before pupils proceed to secondary school. Pupils in these years are usually between 10 and 12 years old, with some early or late pupils aged 9 or 13. Studies have highlighted the importance of focusing on bystander intervention at a pre-adolescent age, since children of that age are yet less likely to be influenced by group norms in contrast to adolescents (Killen et al., 2013; Willems et al., 2024). Salmivalli et al. (2021) portray that effectiveness of bullying interventions in terms of reduced bullying behavior was shown to be most effective at pre-adolescent age. This means that encouraging positive bystander responses and training intervention skills, makes pupils more resilient to go along or accept bullying behavior, even if the bully is someone with higher status of the ingroup. In the Netherlands, a mixed school is defined as a school with approximately between 25 and 80% pupils with a non-Western migration background (Herweijer, 2008). This definition of a mixed school is applied to this study as well. A percentage lower than 25 decreases the chance of teachers having experiences with racism or racist bullying among pupils. With at least 25% we can assume that there is sufficient cultural diversity in a school, and as a result intercultural contact among pupils (Bayram Özdemir et al., 2018; Walton et al., 2014).

No demographic data concerning race or ethnicity is publicly available for schools in the Netherlands. Therefore, in the selection of schools, the diversity of the population of the geographical location of the school was taken into account. Information about the diversity of the population was gained online through the Central Statistical Office of the Netherlands. Schools located in an area with a diverse population were predominantly located in urban areas.

Table 1 shows an overview of participants’ teaching experiences, gender and race. Participant 2 was a primary school teacher of a different grade currently but had 8 years of teaching experience in grade 7 and 8. Participant 7 was currently a primary school teacher of grade 7 and 8 across different schools and accordingly did not have one permanent class. Participating teachers had teaching experiences in primary schools between 2 and 31 years, and teaching experiences in grade 7 or 8 between 1 and 20 years.

**Table 1:** Demographics and characteristics of participants.

Respondents	Gender	Race	Teaching experience primary school	Teaching experience grade 7 and/or 8
R1	Female	White	20	12
R2	Male	White	6	1
R3	Male	White	31	8
R4	Female	Black	2	2
R5	Female	White	22	20
R6	Female	White	14	9
R7	Female	White	25	2
R8	Female	White	25	18
R9	Female	White	31	20

Traag (2018) shows that 3% of primary school teachers in the Netherlands have a non-Western migration background. The percentage of primary school teachers with a non-Western migration background is significantly lower than the actual population of the Netherlands with a non-Western migration background (Traag, 2018). Besides that, about 80% of primary school teachers are female (Traag, 2018).

Teachers were approached and recruited by phone and/or e-mail via schools and the professional network of the research team. Of the nine participants, three were recruited through the professional network of the research team, one via an online call for participants within the academic institution of the research team, and the remaining five by cold-calling schools throughout the Netherlands.

### Data collection

Interviews were conducted in Dutch, with an average length of 42 min and interviews ranged between 27 and 55 min. In two cases participants were colleagues at the same school, therefore the interviews were conducted on the same day immediately after each other to prevent any influence of possible given answers. Six interviews were conducted at the end of the school year between May–June 2023 and three in October 2023 at the beginning of the new school year. The gap between the first six interviews and the latter three interviews was about 3 months. Teachers did not receive any monetary compensation for their participation.

### Procedure

After receiving verbal information about the study and expressing willingness to participate voluntarily, teachers received a digital version of the information letter and informed consent via e-mail. Interviews were scheduled at a location of choice by the teacher, which in all cases was the working place of the teacher. The interviews were conducted predominantly in a classroom. Seven interviews were conducted in a closed room without the presence of others. Interviews 8 and 9 were conducted in a more open space, with pupils and a teacher in the background who did not interrupt or participate in the interview. Appointments were mostly made after school time, or during school time when the teacher was free from teaching duties. Just before the interview, the teachers received a printed version of the participant information sheet and informed consent form. The informed consent form informed participants about their anonymity, data protection and data storage. Besides that, the letter stated that participants could withdraw their participation at any given time without any consequences. The informed consent form was signed prior to the interview. Before the interview started, the interviewer asked for consent to have the interview recorded and guaranteed anonymity. After the interview, an information sheet was shared with more information about school programs and lesson plans about racism or racist bullying. Interview recordings, transcripts and signed informed consent letters were stored on the servers of the Open Universiteit.

The interview questions were informed by previously mentioned literature in the introduction on teachers’ approaches in relation to racism and racist bullying. Within this literature, personal and environmental factors, which influenced teachers on the topic of racism or racist bullying, were identified that fed into the development of the interview topic guide. The interview topic guide was developed by an interdisciplinary research team with backgrounds in psychology, educational sciences and humanities. See supplementary files for Appendix 1 the topic guide with selected determinants and interview questions, and Appendix 2 for the coding tree.

### Data analysis

All interviews were audio-recorded with a voice recorder, transcribed verbatim and eventually coded in ATLAS.ti. (version 23). The data was independently coded by three researchers (KS, TV and NW). Thematic analysis was chosen as a method to identify and analyze codes and themes and report patterns from the data (Braun and Clarke, 2006; Verhoeven, 2023). The thematic analysis entails analyzing experiences and perceptions of individuals in a given context (Verhoeven, 2023), in this case of primary school teachers in an educational setting. Data was analyzed in different phases, which started with an exploratory phase of coding and constructing themes based on the topic guide. Personal and environmental factors from the topic guide were reviewed against the data for the selective coding of fragments. After coding the first two interviews a code book was developed which was used for the remaining interviews. During analysis this code book was adjusted in agreement of the coders, if this was found necessary. This exploratory phase resulted in the phase of reduction whereby themes were organized and revised. By continuously reviewing and comparing codes the data was constantly read and re-read. The last phase was the reflection phase, in which structures were determined through themes in relation to each other and to the research questions (Verhoeven, 2023). Any inconsistencies after analysis were resolved by mutual agreement of the coders. The first five interviews were coded by KS and TV with Krippendorf c-alpha inter-coder agreement (ICA) of 0.841. The last four interviews were coded by KS and NW with an ICA of 0.791. Both outcomes indicate an acceptable level of reliability of the coded data since there is sufficient agreement between the coders (ATLAS.ti Scientific Software Development GmbH, 2023).

New information was gathered based on gaps found during the analysis (Verhoeven, 2023). In this study, the teachers’ racial identity in relation to racism was a notable additional topic that was brought to attention by participants. Literature seemed to stress the importance of teachers’ racial identity and how it affects approaches toward racism (Johnson, 2002; Perez, 2022; Sijpenhof, 2021). From the seventh interview, the question about the teachers’ racial identity was included in the topic guide. Since racial identity was brought up by a few participants in the first six interviews, the remaining three interviews, whereby questions about racial identity were included, did not reveal any new information and was considered to be sufficient to answer the research questions of the current study. A question about colorblindness was dismissed after the third interview to avoid any further confusion, since two out of three participants did not understand this concept. Colorblindness, or as Annamma et al. (2017) prefer to call it, color-evasiveness, is the purposeful denial and refusal of addressing race and racism. After nine interviews the information received from the teachers seemed sufficient to answer the research question(s), additionally some answers became repetitive and no new insights were found from the interviews concerning the research question(s). Data saturation seemed to have been reached, however it must be taken into consideration that future research might benefit from including more racially diverse respondents. The following themes emerged out of the data: teachers perceived barriers in addressing racist incidents, the (un)intentionality and (un)awareness of pupils, racial identity, differentiation between racist and interpersonal bullying, racism and culture, coping with racist incidents, creating a safe class climate, school climate and educational material and tools.

### Positionality

The positionality of the researcher who conducted the interviews, a woman with a mixed-race (Black and White) background, are taken into consideration. This position might have influenced how participants interacted with the researcher, and what they therefore included and excluded in their answers (Castagno, 2008). White people discussing racism exclusively among each other allows a more unfiltered view instead of giving politically correct answers in the presence of non-White people (Sijpenhof, 2021). As a matter of fact, non-White researchers purposefully decide to have White researchers interview White participants about race or racism, to avoid hesitation or reservations of answers (Morris, 2022). In this study, one participant explicitly mentioned the uncomfortableness during the interview of discussing racism as a White person and being interviewed by a mixed-race researcher. Even though uncomfortableness was experienced by this participant, and perhaps even by more participants, it was perceived that the positionality of the researcher did not vitally influence the given answers by the participants. Besides the influence of the researchers’ racial identity on the participants, the analysis and interpretation of the data depend on the researchers’ personal background as well (Henze et al., 1998). The social identities of the researcher should be considered in relation to the research context (King et al., 2023). To minimize bias, the data was coded by the researcher with a mixed-race background (KS) and two White researchers (TV and NW).

## Results

The results of this study demonstrate that teachers perceived a few barriers concerning racist bullying, such as external influences on pupils. Most of them did not differentiate between interpersonal and racist bullying. Besides that, teachers often believed in the innocence of their pupils. Discussions about racism seemed to be more about the cultural diversity of pupils rather than racism per se. Schools likewise seemed to emphasize cultural diversity and rarely included racism in their discourse.

### Teachers perceived barriers in addressing racist incidents

According to most teachers, racist bullying did not seem to occur very frequently, as they saw bullying more as a structural phenomenon. Teachers rather indicated that they more often encounter racist comments or expressions, which hereafter will be referred to as *racist incidents*. In general, teachers expressed that it is challenging to address racist incidents. One of these challenges is first and foremost visibility. Teachers mentioned the difficulty of keeping track of racist incidents because they frequently happened out of sight and were rarely visible during class. A teacher mentioned that racist incidents would happen, for instance, “in the hallway, during physical education and especially when playing outside” (R1). They would also occur at extracurricular childcare or when they were “roaming through the neighborhood” (R3). Teachers therefore often got notified afterwards by the victim, bystanders and on rare occasions, by the parents. A second challenge teachers seemed to face was determining if an incident is racist or not and, consequently, determining its severity. A few teachers found it hard to determine when something is teasing or when it can actually be considered as a racist incident. As a result, these teachers found it challenging to determine how much attention to give to a possible racist incident. Additionally, if an incident was interpreted as racist, the response of the victim could be a decisive element in determining its severity. For example, if the victim started laughing as response, it made it more challenging for the teacher to intervene. “But is it laughing because you like it, because you think, let me just laugh along but I actually do not like it?” (R7). Another teacher elaborated by stating that victims have a tendency to laugh racist comments off.

I also try to explain that if it affects you, if it hurts you, you should speak up and not just laugh about it, because that happens a lot (…), but it actually did affect them, so they are actually not really sincere toward their own feelings. (R5).

The third aspect teachers found challenging in addressing racist incidents was the way pupils were influenced by external factors. The predominant external factors mentioned were the pupils’ families, traditional media and politics. One teacher explained how family influences contributed to a child’s racist beliefs.

There was also one person who could say quite nasty things about others. You could notice that his grandpa is active in local politics, but then in a party which is rather, you know (…) with some kids yeah, you just feel that’s their upbringing. They do not know any better. (R8)

Another teacher confirmed this thought of normalization and stated that she often hears the “father or mother talking” of the children who express racist comments “because in their home situation, it is normal” (R9). She therefore believes that parents actually need to be educated. Additionally, she mentions the influence of local politics on children in their expressions such as, “They should not be building that, because it’s not for our people” (R9) when her class was passing by a mosque under construction. Another teacher made a similar statement about how the traditional media causes racism and how this influences the pupils’ perceptions since people from different cultures “are being portrayed in a negative light” (R5).

While teachers believed that pupils’ racist beliefs and expressions were influenced by external factors, some teachers also thought that some pupils struggled with these thoughts, since they might not agree with this racist point of view and are therefore “divided” (R9). “Um, the children who get this [racist point of views] from home, (…) they live in two worlds (…). So I understand that it is difficult for those children” (R8). This inner conflict pupils can have also seemed difficult for these teachers to cope with. “So sometimes I find it very difficult to get through to these children because they do give socially desirable answers (…) So I think that’s still a very tricky one. I do not have a very good strategy for them yet” (R1). Another teacher addressed how pupils might find it difficult to voice another opinion toward their parents’ racist point of view. “When you hear those [racist comments] at home, of course, it’s I think, you have to stand firm in your shoes if you want to say to your dad, I think differently about that” (R6).

### The (un)intentionality and (un)awareness of pupils

Most teachers argued that pupils were often unaware of the severity of their actions, and that pupils mostly acted unintentionally. As one teacher puts it “Well, my experience is that children often do not realize that they are really making [racist] comments, (…) if you engage with the kids and really explain things to them then often they do not know what they are saying either” (R4). While most teachers believed that pupils were often unintentional and unaware of racist comments, one teacher believed that pupils were also unaware of the consequences. “Well, they (pupils) always laugh a little about it [racist comments] (…). Well then you just think, do you know what you are saying and what it can cause? Actually, they do not know that about each other” (R5). This teacher also believed that these pupils were not capable enough of determining the consequences of their actions “because it’s just a joke” and “their perception in their world is still maybe just a little too small to see the implications of that [racist comments]” (R5). Only a few teachers believed that pupils were very much aware of what they were saying.

They want to bully, they are looking for something, whereby you of course hurt someone, and that’s often it’s, for example your origin or religion or something like that, so that’s what they are looking for. In that moment they look for something to hurt someone. (R6)

### Racial identity

From the first six interviewed teachers, three teacher were aware of their own racial identity. After these six interviews the remaining three teachers of the sample were asked about their racial identity in relation to racism during their interview. Most of these teachers had a self-reflective approach and stated that not being able to relate to racism as a White person, might be challenging and complicated for them to really understand and determine the severity of racist incidents. “I cannot relate to it [racism]” (R5). Another teacher stated the same lack of experience.

I will probably not always recognize it [racist bullying], just because I do not know such experience myself” (…) You cannot feel it, if you have not experienced it, I guess. Of course by hearing or reading about experiences you can put yourself in that situation a bit, but never for real. I do not think I can say how someone feels who has been racially discriminated, and I really find it disturbing, and I really find it, I think I can imagine how bad it can be, but I can never [imagine this] completely. Just because I did not have such experience myself. So that makes it challenging of course. (R6)

One teacher stated that she felt it was not her place to lead the discussion about racism as a White person, because of the lack of lived experiences.

So I am talking about something [racism] of which I have no experience of, so it feels a bit like, take a step back, you know, this is not, this should not be your stage. So it feels, it just feels complicated. (R1)

This feeling, however, did not stop her from proactively discussing racism in class. Another teacher was also aware of her own racial identity as a Black teacher. She expressed how she senses that pupils, who do experience racist incidents, are pleased to have a teacher who can understand the severity of these incidents and therefore addresses these. Other teachers did not mention anything about their racial identity or found it difficult to describe theirs. In a few cases they related their racial identity rather on how they perceive and react to others, for example by stating to have a positive attitude toward immigrants and refugees.

### Differentiation between racist and interpersonal bullying

Almost none of the teachers found it necessary to distinguish between racist and interpersonal bullying, or they doubted if different approaches were necessary. One teacher clearly noted that all forms of bullying are hurtful and can have a big effect on a victim.

Bullying to me is bullying regardless of its form, and I do not want that, because it’s just something that someone just, it just hurts and it can just have a big effect on someone. It’s also often the case that if you have been bullied from a young age and you get older, it can still hurt you, whether it’s because of skin color or whether it’s something about someone having one leg longer than the other, it does not matter, it’s bullying and it’s not nice. (R4)

Another teacher made a similar statement. “If you are being bullied, it does not matter for which reason, you easily feel like you do not belong” (R3). One teacher found it difficult to distinguish between interpersonal and racist bullying. She was not decisive about whether racist bullying is worse than interpersonal bullying or not. Another teacher also mentioned that she addressed racist incidents just as interpersonal incidents. “So of course I say, I do not think what you are saying is okay, but I also say that because they say they are wearing an ugly t-shirt or because it’s gay” (R7). This also seemed to be the same approach for another teacher who indicated that there is no differentiation between the consequences of racist and interpersonal bullying. “The approach is the same, so whether it is racist or not” (R9). Another teacher, however, was unsure about a different approach. “I also do not know if it [racist bullying] requires a different approach than just [interpersonal] bullying” (R1).

### Racism and culture

Racism did not seem to be a topic at any of the teachers’ training. Still, most teachers expressed feeling competent and comfortable enough in discussing racism in class and addressing racist incidents, due to their teaching experience. Feeling competent was also due to the fact that “pupils’ are often very open and honest” (R1) which would make discussions about racism easy. A few teachers mentioned that racist incidents were often the trigger for discussing racism or when it was occasionally mentioned in educational material. However, when teachers claimed to discuss racism in class, most of them could not specify or elaborate on examples. Generally, they seemed to talk about cultural diversity and ethnicity, rather than explicitly discussing racism. Thus, discussions about racism itself and the possible consequences were very few.

Teachers explained their focus on teaching cultural awareness and knowledge about different cultures by referring to the idea that the current society that pupils live in has become more multicultural. Due to a belief of multiculturalism only being a recent phenomenon, a few teachers claimed that racism too seems to be more of a recent problem. One teacher explicitly believed that racism “did not exist” (R9) 30 years ago and stated that it was a “no-go” (R9) to talk about in previous years. Another teacher mentioned that racism “was not discussed” (R1) 20 years ago in society, but also not in class.

### Coping with racist incidents

Despite most teachers talking about cultural diversity rather than racism, some did believe that racist incidents occurred due to the pupils’ lack of knowledge about different cultures. As one teacher stated “I often say, these kind of things [racist incidents] evolve due to fear, not knowing [about the culture] (…). Why does someone do this? Can it be because of their cultural background?” (R5). When teachers tried to address racist incidents, engaging in conversation seemed to be the most mentioned strategy by teachers. Most of the teachers’ reaction toward the victim, bystanders and bully also depended on the context, and their character. This means that some teachers purposefully addressed the racist incident in class while others had a private conversation with the victim and/or the bully. Engaging in conversation with the victim, bystanders and bully was the predominant reason why most teachers perceived themselves as competent enough to address racist incidents. Even though teachers addressed racist incidents and felt competent enough in doing so, most of them did not further clarify what was racist about the incident and what negative implications it could have for the victim. Teachers did mention, however, that they were willing to learn more skills to address racist incidents. Skills they found important in addressing racist incidents were mostly communication, such as listening and having an open attitude toward the victim but also toward the bully. Being fearless and having empathy were other skills teachers found important, but also knowing the character and personal situation of the bully, and persuasion, by being able to convince the bullies of their wrongdoings. Important strategies that were mentioned were, being consistent, setting boundaries, being understanding toward the victim and bully, and being sensitive about the racist incident.

#### The victim

Overall teachers had a very supportive attitude toward the victim. Almost all teachers mentioned the importance of the victim feeling heard and understood. While trying to be approachable, a few teachers did believe that victims often feel afraid and ashamed to report the bullying victimization. How teachers reacted toward the victim varied. Some teachers tried to attend to the victim first to get the first impression and understanding of the situation, whereas others attended to the bully first. Engaging in a conversation with the bully first, however, could create an unsafe feeling toward the victim according to one teacher.

I also think that it is important, that you do not have a conversation with a bully like that right away, because well, sometimes that just feels unsafe right away. So first the story needs to be heard, so that they [victims] are safe coming to you, as a teacher. (R1)

Teachers generally want to make clear that the behavior of the bully is unacceptable. The previously mentioned teacher adds, that not only is a racist incident unacceptable, but the victim “must know that it is not their fault” (R1). Teachers additionally found it important that the victim decided on closure. This could be accomplished by asking the victim if the approach of the teacher was sufficient or not. Ignoring the racist incident and not attending to the victim was mentioned as an ineffective strategy according to the majority of teachers. Another ineffective strategy was putting the victim on the spot in front of the group, as one teacher expressed.

I can imagine that some people might have that idea because then you might think that action is taken immediately, but I have always learned that it is important to first talk with children individually about such a situation, whether they are at peace with it, because after all, they are victims in such a situation, and you do not want to put them on the spot or showcase them. (R2)

Teachers expressed that degrading the racist experience of the victim is an ineffective strategy as well. One teacher mentioned an interaction he had with a colleague.

Unfortunately, I have experienced colleagues who say, “That’s not so bad,” but you do not know what it means for such a child. You can never say: it’s not so bad, because a child like that, that’s dealing with it, that’s perhaps dealing with it a lot, then you say it’s not so bad. I cannot determine whether it’s not too bad (…), maybe I used to say that too, but I became much more aware of that. If a child is sad or is hurting inside, then I’m not the one who has to say: gee, do not be so worried, it’s not so bad what has been said. (R3)

#### Bystanders

Most teachers tried to include bystanders, yet they seemed to get less attention than the victim. In fact, one teacher mentioned that bystanders were a “blind spot” (R2) to him since he usually did not involve them. Two teachers noted that their school is working with a method to actively involve bystanders. If teachers included bystanders, some only involved those who witnessed the racist incident, whereas others applied a whole class approach to address the racist incident. To what extent bystanders were involved usually depended on, for example, how big the teacher thought the issue was, where it happened and if it was a structural pattern. Additionally, involving bystanders depended on whether the teachers thought the class had a safe climate, and how they expected the pupils might respond. One teacher stated that it is better not to give racist incidents too much attention by involving bystanders, because then you might make it bigger than it is, and “everything you pay attention to grows” (R7). Another teacher, however, discussed how not making things bigger than they are will not work for racist bullying, because you have to address it, also openly, “otherwise you will not get it eradicated” (R3).

#### The bully

Most attention after a racist incident seemed to go to the bully, as teachers disapproved of such behavior and wanted to create awareness about the behavior being unacceptable toward the bully. Metaphorically, two teachers did this by crumpling a piece of paper and showing that no matter how much you try to smooth out the paper, it will never have its original texture again, just as a victim of racist bullying. Besides that, while some teachers believed punishment was an effective strategy, other teachers disagreed.

Well punishment, of course they [bullies] will keep their mouth shut, maybe because they might get expelled. But I guess, well their thoughts are not different (…). I am not sure if it is really, well, the solution, because you do not really completely, well you do not stop the [bully’s] thoughts. (R6)

A few teachers doubted if punishment would be an effective strategy, and thought it might be a solution for repetitive behavior only. Some also believed that “victims might like to see a consequence” (R6), such as a punishment, to “feel heard and seen by the teacher” (R8). Nevertheless, a few teachers did punish the bully after a racist incident, also because this aligned with their school policy.

Other strategies teachers found ineffective were putting the bully on the spot, not being consistent toward the bully, being too angry, and showing disapproval right away without giving any further explanation. Rather than being angry, creating understanding for the bully was found to be an effective strategy, especially by explaining why the teacher disapproved of the behavior. Most teachers also found it an effective strategy to approach the parents of the bully if, for example, they found the incident too severe, or if the bully did not respond as expected while being addressed about the incident. Some teachers also found it important to keep on providing safety for the bully by approaching the bully with an open attitude and by not being judgmental, regardless of the bully’s behavior. This is also due to the fact that these teachers had a victimizing approach toward the bully since they believe that “often the bully has also been a victim” (R5) and “someone like that should also be helped” (R7).

### Creating a safe class climate

While some teachers believed that they need to ensure a safe space for bullies, teachers do state that racist incidents lead to an unsafe environment. This feeling of being unsafe could then affect the learning capabilities of pupils negatively. Most teachers believed that it is the responsibility of the teacher to create a safe environment for their pupils. If there is not a safe class climate “then you are not going to accomplish much” (R4). Teachers found it hard to explain how a safe class climate could be created, it is rather something you just try to create “by, for example, having conversations” (R6), or “exposing yourself as a teacher” (R9). The latter teacher referred to exposing personal experiences and insecurities.

#### Group dynamic

The group dynamic was mentioned by a few teachers as an aspect that influenced the class climate. This dynamic depends on how the pupils interact with each other, but also the position they feel they have within the group. Furthermore, the hierarchy of the pupils also influences the class climate. Some teachers believed that pupils are often afraid of the bully and do not always dare to speak up when a racist incident occurs.

I believe that pupils, the children who dare to say things like that, racist bullying, that they have a certain position in the group, which is quite high. So I also do not think that all children dare to say something about that the moment it happens (…). I think kids do not feel they are in that position to speak up. (R2)

#### Teacher-pupil relationship

Another important aspect teachers mentioned for a safe class climate is the teacher-pupil relationship. Some teachers found it important to have good individual relationships with the pupils. They believe it is necessary to know the characters and background of pupils, to know the position of the pupils within the class, but also to know their home situation to some extent. Additionally, the cultural background was considered of importance as well. “You must also consider, do they [pupils] come directly from another country? Or are they born here? Are they second or third generation? Extremely important to know this!” (R3). This was also mentioned by another teacher who considered other backgrounds as well.

(…)We have children who are refugees and have only been here since two or three years. We have expats who decided to stay longer or permanently. So you have a lot of different inputs from different children with different cultural backgrounds. (R5)

This knowledge about the pupils, according to these teachers, can create a better understanding of them, and can also facilitate how teachers respond to pupils depending on the approach they think the pupil might need. Teachers likewise mentioned that a safe class climate is necessary for children to share personal experiences and opinions.

### Environmental factors

#### School climate, educational material and tools

Schools mostly did not pay much attention to racism in the curriculum. One teacher said that his school is not really active in approaching racism as a stand-alone topic. “We as a school have not actually been looking for, I have to admit, for books or things [about racism]” (R3). As previously mentioned, teachers considered it important to emphasize differences of pupils’ cultural background. Schools seemed to focus very much on acknowledging different cultures. They organized festivities and excursions and highlight cultural holidays. Some schools even actively celebrated different cultural holidays. Schools also developed different tools, such as videos, to create cultural awareness among the pupils. Teachers not distinguishing between racist and interpersonal bullying can be seen as a reflection of the school climate. Even though teachers do encounter racist incidents, such incidents are not being discussed in the school policy. Racism did not seem to be a stand-alone topic, neither in the school policy nor the bullying protocols. Teachers and their colleagues also rarely seemed to discuss racist incidents with each other. “Let me think, well I think it is kind of, well I do not have like an example right now” (R6). They did not remember any discussed incidents “Well nothing I can remember of” (R7), or assumed their colleagues would mention similar experiences. “Well actually [I would hear] kind of similar things. I do not think it [racist incidents] happens that often, fortunately. It does happen in different groups, but not really structural” (R8).

#### Tools and needs for discussing racism

Most teachers stated that racism is a topic that is often not a stand-alone topic in the school curriculum, or it is not part of the curriculum at all. Racism seemed to be mentioned in educational material coincidentally. Some teachers only addressed racism when it was mentioned in the educational material or after a racist incident occurred. One teacher pointed out that she would not initiate this conversation on her own. “It does not really happen very often that I think, well, let us pick that up now [racism]. It’s always linked to something, though, because either something has happened, or [it’s in the material]” (R7). A few teachers, who did pro-actively discuss racism itself in class, or were influenced by a colleague to do so, regardless of whether racism was mentioned in the material, did not shy away from searching for extra material or information. These teachers also seemed to try to link racism with the current Dutch society “to make the subject a subject to discuss” (R1).

Tools about racism seemed to help as a conversation starter and to engage discussions about racism. Teachers had some suggestions for other tools they would like to use for discussing racism in class. Mostly teachers mentioned the need to have examples, real-life experiences, in the form of stories or videos, for pupils to understand the consequences racist bullying can have on victims. Other needs were tools whereby pupils will be able to relate to what racist bullying feels like. Another need that was mentioned was having more historical knowledge and background information about racism. Some explicit examples were mentioned, such as case studies and digital tools, like games and information which can repeatedly be used and referred back to, and can be a conversation starter throughout the school year. This could be in the form of cross-group material, so parallel classes could treat the same subject with the same approach, or even cross-school material to engage more schools into the discourse of racism. Teachers also expressed the need for tools to recognize racism on time, as one teacher explained, “I also notice that things still sometimes happen behind my back which then escalate, because I do not recognize it on time, and I would perhaps like to get some more guidance for that” (R2). Another teacher mentioned the need for self-reflective tools for pupils, whereby pupils can discuss both the side of the bully and the side of the victim. A “parental guide” (R8) was also mentioned since there are some parents that hold racist beliefs which they pass on to their children. For the most part, teachers mentioned tools which can be used for a pro-active approach.

## Discussion

This study explored teachers’ responses to racist bullying and discussing racism in class in Dutch primary schools. Firstly, we explored teachers’ attitudes and beliefs about racist bullying among pupils and about teaching racism. Attitudes and beliefs of teachers were expressed throughout the various themes of the result section and will also be addressed in the discussion of the other research questions. In general, the results demonstrate that according to most teachers, racist bullying did not seem to occur very frequently, as they saw bullying more as a structural phenomenon. Teachers rather indicated that they more often encounter racist comments or expressions. Mostly, teachers believed that pupils were unintentional and not fully aware of their racist expressions. Additionally, teachers believed that interpersonal and racist bullying were equal. Therefore, their attitude toward racist incidents was the same as toward interpersonal incidents, which corresponds with the approaches of most schools toward these forms of bullying. Teachers also believed that it was necessary to create a safe class climate for their pupils, emphasizing teacher-pupil relationships for appropriate responses, and for pupils to dare to share personal opinions and experiences. This finding corresponds to the findings of the study by Wansink et al. (2023), who highlight the importance of creating a safe classroom for discussing controversial issues. Discussions regarding racism mostly arose due to a reactive response after a racist incident, or if racism happened to be addressed in educational material. This is similar to the findings of Priest et al. (2016) and Daniel and Escayg (2019), demonstrating that most teachers discuss racism as a reactive rather than a pro-active approach. While teachers mentioned that they talked about racism in the classroom after a racist incident occurred, it seemed that they primarily talked about cultural diversity and not racism per se. Teachers expressed that racist incidents were unacceptable and mentioned a few challenges in addressing these, such as determining the severity.

Secondly, we aimed to gain insight into teachers’ experiences of and reactions toward racist bullying among pupils. When reacting to racist incidents, teachers mostly engaged conversations with the bully, victim and sometimes the bystanders. Teachers expressed a supportive attitude toward the victim, by being approachable and letting the victim feel heard and understood. Some teachers involved the whole class after a racist incident, while others only involved the bystanders who witnessed the incident, whereas a few teachers did not involve bystanders at all. Teachers had a disapproving attitude toward the bully and intended to make clear that such racist remarks were unacceptable. Teachers also believed that external factors influenced pupils’ beliefs and attitudes about racism. They described how parents’ racist attitudes and beliefs influence the racist attitudes and beliefs of their children. Similar findings have been expressed in various studies (Bartoli et al., 2016; Curran et al., 2023; Sinclair et al., 2005). Not only racist attitudes and beliefs of parents can be harmful to their children, but also their false beliefs about racism, such as it not existing, or it not being important (Bartoli et al., 2016). Boutte et al. (2011) mention how adults in general play a vital role in the misconceptions and racist behavior of children. Besides parental influences teachers believed that some pupils might experience an inner conflict with their parents’ racist expressions. The results of this study show that teachers found it challenging to cope with these influences and the inner conflict pupils might have.

Another common feature of teachers’ reactions to racist incidents was that they rarely acknowledged there was a racial motive behind pupils’ bullying behaviors, as they believed pupils were mostly innocent and unaware of their actions. These findings are similar to findings of the study by Pearce (2018), who demonstrated that teachers focus more on individual intentions rather than on the outcomes of racist incidents. Daniel and Escayg (2019) correspondingly illustrate that teachers portray their pupils as innocent and unaware of race and the racist nature of their comments. Pupils may not understand the negative implications of racism, as one teacher explained in this study, but research shows that they are aware of racial differences and its power dynamics from an early age (Boutte et al., 2011). Being reluctant to acknowledge a racial motive behind children’s behavior is a way in which the severity of racism is underplayed in the school context (Jones et al., 2023).

Thirdly, we explored what tools and skills teacher need to approach racist bullying among pupils, and to teach about racism. Teachers found communication one of the most important skills for addressing racist incidents individually or in the group. This communication entailed listening skills and an open attitude toward the victim and the bully. The knowledge teachers had about racism seemed to be restricted. This consideration is reflected by beliefs such as racism only being a recent problem or that it did not exist many years ago. Teachers lacking an understanding of racism corresponds with findings of the study of Wilson and Kumar (2017), who point out that teachers often misunderstand racism and see it only as individual acts rather than an institutional problem. Teachers having limited knowledge about racism is not surprising since none of the teachers of this study learned about racism during their teacher training, or were provided with any further training. Daniel and Escayg (2019) found similar results regarding the training of early childhood educators, who educate children before primary school. Their training did not include any or very limited content about race or racism, which similarly to this study, limited teachers’ ability to effectively address racist incidents.

The schools the teachers of this study worked for, did not provide any further knowledge or tools about racism, except for the limited information the teaching resources supplied. This indicates that, unless the teacher independently searches for materials to increase their knowledge, their knowledge on racism and addressing racist incidents will be limited, which is comparable to the findings of Brielle Harbin et al. (2019). A few teachers of this study who did want to learn and teach more about racism, had to do this on their own initiative. The lack of institutional support for teachers of this study does not stimulate them to pro-actively or even reactively discuss racism in class. This finding corresponds to findings of Perez (2022) and Phillips et al. (2019), who demonstrate that a lack of institutional support can be a hinderance for teachers to discuss racism. The results of this study further show that teachers did express a need for tools about real life experiences and examples of racism with, for instance stories and videos, to discuss racism within class.

With our final research question, we investigated the perceived competence of teachers on how to deal with racism. Most teachers in this study perceived themselves as comfortable and competent enough in discussing racism in class, however, their perceived competence seemed to differ from their reported behavior. Racism itself was seldom discussed. Instead, teachers seemed to discuss cultural differences of pupils and cultural diversity, which seemed to be related to the teachers’ belief that racist incidents occurred due to the lack of knowledge pupils have about different cultures. The finding of teachers focusing on cultural diversity is in line with various studies that highlight how teachers tend to focus their discussions on ethnicity and culture instead of discussing race or racism, and that teachers may even use these terms interchangeably (Castagno, 2008; Daniel and Escayg, 2019; Johnson, 2002; Pearce, 2018; Priest et al., 2016).

A few teachers in this study found it challenging to determine and understand the severity of a racist incident, which is similar to findings of a study by Hurtubise et al. (2020), who showed that even if pupils report racist behavior a teacher can have difficulties in identifying the racist behavior. Most teachers, however, perceived themselves as competent enough in addressing a racist incident if it did occur. Teachers clearly disapproved of racist expressions and made clear that such expressions were offensive and hurtful. Yet teachers did not distinguish between interpersonal and racist incidents and approached both incidents the same way, and often did not communicate what was racist about the incident. Thus, there seemed to be a discrepancy between their perceived competence and their behavior toward addressing racist incidents. Not distinguishing between interpersonal and racist incidents shows that their perceived competence might relate more to general conflict resolution skills than conflict resolution skills for racist incidents. Dunning et al. (2003) mention that preconceived notions about certain skills can cause people to misjudge their actual performance. In this study teachers might have had a preconceived notion about their general conflict resolution skills and therefore perceived themselves as competent enough in addressing racist incidents. Teachers not distinguishing between interpersonal and racist incidents also indicates that they might underestimate the severity of the negative implications that racism has on victims. Shah and Coles (2020) argue that it is important for teachers to highlight the negative societal implications racism has on victims when discussing racist incidents.

Previous research has found that the racial identity of the teacher also influences his or her attitudes and approaches toward pupils from different cultural backgrounds (Tettegah, 1996). In this study the results suggested that some teachers were aware of their own racial identity but most teachers were not. Most White teachers did not mention anything about their own racial identity in relation to racism, or when asked about their racial identity were hesitant and found it hard to describe theirs. This finding corresponds with those of other studies by Picower (2009) and Vachon (2022), who also found that White teachers were often unaware of their own racial identity. Various studies highlight the importance of teachers’ acknowledging their own racial identity particularly in relation to society and its power dynamics, and in relation to the intersection of privileges and racism (Picower, 2009; Priest et al., 2016; Solomona et al., 2005; Vachon, 2022). Shah and Coles (2020) stress on the importance of teachers reflecting on their own racial identity, in terms of positionality and privilege, as one of the first steps when attending to racist incidents.

## Limitations

The current study is subject to some limitations. The study involved a convenience sample of teachers that is thus not representative of the general teacher population in the Netherlands. This convenience sample also resulted in a lack of racial and ethnic diversity among the sample of teachers. Therefore, it would be of benefit to purposefully include more teachers with a diverse racial and cultural background concerning future research on racism and racist bullying. There was no compensation for participating teachers, therefore their own interest in the topic was the key motivator of involvement (Jones et al., 2023). Results are based entirely on teachers responses to the questions, there were no classroom observations to support their statements.

## Implications

The results of our study suggest that adequate training on racism and tackling racist based bullying could be an effective strategy in diminishing teachers’ discrepancies between their perceived competence and behavior of discussing racism and attending to racist incidents. This calls for a need to provide teachers with more appropriate tools to increase knowledge, awareness and skills (Willems et al., 2024). Providing these could help teachers understand the negative impact of racist bullying on children’s development and feelings of belonging. Knowledge and skills in combination with the appropriate tools are also necessary to apply a pro-active approach to discuss racism in class instead of discussing racism incidentally or as a reactive approach after a racist incident. A pro-active approach also entails teachers critically reflecting on their own racial identity in relation to society and critically reflecting on school methods, materials and policies concerning racism, which can be used as a preventive method to avoid racist incidents (Boutte et al., 2011; Vachon, 2022).

On a micro level this study recommends that teachers not only limit conversations to cultural diversity but also discuss racism and its negative implications for victims, to create a better understanding for pupils and to actively stimulate bystanders to intervene in racist incidents (e.g., Willems et al., 2024). On a macro level this study recommends that Dutch teacher training include critical knowledge about race and racism (Alvarez and Milner, 2018) and that long term professional development programs about racism are provided for teachers (Henze et al., 1998). Additionally, this study advocates for schools to integrate discourses about racism into the existing curriculum (Boutte et al., 2011). Future research is recommended on examining Dutch teacher training and Dutch educational material concerning the discourse of racism.

## Conclusion

This study shows that teachers emphasize a safe class climate and clearly disapprove of racist incidents and address these. In addressing racist incidents and discussing racism discrepancies were found in the teachers perceived competence and their behavior. While responding to racist incidents teachers did not seem to communicate why the incident was racist. Additionally, teachers seemed to discuss cultural diversity rather than racism. The lack of tools and further training on racism in schools as well as a lack of education on racism during teacher training, highlights the urgency to provide teachers with further training and tools to teach pupils about racism and its negative implications. Besides that, awareness and a critical reflection of one’s own racial identity as a teacher would help in navigating this challenging and sensitive discourse of racism.

## Data Availability

The original contributions presented in the study are included in the article/Supplementary material, further inquiries can be directed to the corresponding authors.
