# Supplementary material for: Teachers’ responses to racism and racist bullying in Dutch primary schools
Source: Front Psychol. 2025 Jan 23;15:1393719. doi: 10.3389/fpsyg.2024.1393719 (PMC11798949; doi:10.3389/fpsyg.2024.1393719)
Supplement: Supplementary file 1 [file Table_1.pdf]

Appendix 1 - Topic Guide Semi-structured Interviews

Italic text/questions are optional and depend on previously given answers.

**Determinant**	**Questions**
**Teachers' experiences**<br><br>**Teachers' beliefs**	According to the safety monitor, 11% of pupils in grades 6 up to 8 have experienced bullying sometimes or often in the last two school years (*at least once a month)* (van den Broek et al., 2022). What are your experiences from the past period?
	In doing so, pupils will also have experienced racist bullying. What are your experiences regarding racist bullying?
**Teachers' knowledge about racist bullying**	What is racist bullying according to you?
	How easy do you find it to distinguish between interpersonal bullying and racist bullying?<br><br>*How do you make this distinction?*
**Teachers' experiences**<br><br>**Teachers' beliefs**<br><br>**Teachers' outcome expectations**	Have you ever experienced racist bullying among pupils?<br><br>*How did you feel about it?*<br><br>*Is it discussed in class?*<br><br>Or → How would you handle racist bullying if you saw it among pupils?<br><br>*Optional: give examples*<br><br>*Continue at question:**
**Teachers' beliefs, attitude, experiences and skills towards racist bullying**	How did you deal with it?<br><br>*reaction towards bully*<br><br>*reaction towards victim*<br><br>*reaction towards bystander*
	How do you feel about that afterwards?
**Teachers' perceived behavior of others**	How do pupils who witness racist bullying respond?
	*What have you seen or heard about this from other teachers?<br><br>And from parents?
	How do you handle racist 'jokes' or comments in class?

<table>
<tr><td rowspan="2">Teachers’ skills, beliefs and perceived competence towards racist bullying</td><td>Optional: outline scenarios
reaction towards bully
reaction towards victim
reaction towards bystander
Is it discussed in class/Would you discuss it in class?</td></tr>
<tr><td>What strategies do you think are effective in countering racist bullying behavior?
response to bully
response to victim
response to bystander
What is not effective?
response to bully
reaction towards victim
reaction towards bystander</td></tr>
<tr><td>Teachers’ perceived competence</td><td>To what extent do you feel that you have enough knowledge and skills to handle racist bullying?
(Negative → What could help with this?)</td></tr>
<tr><td>Teachers’ skills</td><td>What skills do you think are important to address racist bullying?
response to bully
response to victim
response to bystander</td></tr>
<tr><td>Teachers’ knowledge about racism</td><td>Announcing transition to racism
Was the topic of racism discussed in your teacher education program?
Yes → How was it discussed?
No → In hindsight, would you have felt the need to have it discussed?
Did you ever receive in-service training on this?</td></tr>
</table>

<table>
<tr><td>Teachers’ attitudes towards different cultures</td><td colspan="3">To what extent do you reflect on other cultures in your classroom?
What is the reason for this?
To what extend does the cultural background of pupils in class play a role in this?
How important do you think it is to reflect on this?</td></tr>
<tr><td rowspan="2"></td><td colspan="3">Do you ever discuss racism in class?</td></tr>
<tr><td>Yes</td><td></td><td>No</td></tr>
<tr><td>Class climate
Teachers’ tools?</td><td>In what ways do you discuss this in class?
Also in relation to the postcolonial past and heritage?
Or: also from a historical perspective?
How are projects around museums/museum objects, monuments, historical sites, traditions/cultural practices used?
How do students respond?</td><td>Outcome expectations</td><td>How would you expect students to react if you were to discuss it?</td></tr>
<tr><td>Teachers’ tools</td><td colspan="3">What teaching materials do you have available to discuss racism?
How do these materials help?</td></tr>
<tr><td colspan="4">The topic of racism appears to be very uncomfortable for teachers to discuss in class (Sijpenhof 2021, Yared et al., 2020).</td></tr>
</table>

<table>
<tr><td>Teachers’ attitude towards racism</td><td>How comfortable or uncomfortable do you find it to discuss this in class?</td><td>Teachers’ attitude towards racism</td><td>How comfortable or uncomfortable would you find it to discuss this in class?</td></tr>
<tr><td>Teachers’ tools</td><td>What helps/would help to make it easy to discuss?</td><td>Teachers’ tools</td><td>What do you think would help to make it easy to discuss?</td></tr>
<tr><td>Teachers’ perceived competence</td><td colspan="3">How competent do you feel in teaching about racism?</td></tr>
<tr><td rowspan="2">School climate</td><td colspan="3">How does your school deal with racism?
And with racist bullying?
Is there anything in the school policy about it?
Does it come up at e.g. teacher consultations?</td></tr>
<tr><td colspan="3">What advice would you like to give other teachers in addressing racist bullying?</td></tr>
<tr><td>Racial identity</td><td colspan="3">How would you describe your own racial identity?
To what extent are you aware of your own racial identity in relation to racism?</td></tr>
<tr><td>Lesson plan</td><td colspan="3">What do you think should be included in the lesson plan? (regarding historical or cultural heritage, and current cultural events, such as e.g. Keti Koti/Ramadan)</td></tr>
</table>
