# Supplementary material for: Teachers’ responses to racism and racist bullying in Dutch primary schools
Source: Front Psychol. 2025 Jan 23;15:1393719. doi: 10.3389/fpsyg.2024.1393719 (PMC11798949; doi:10.3389/fpsyg.2024.1393719)
Supplement: Supplementary file 2 [file Data_Sheet_1.pdf]

Appendix 2 - Coding tree

1. **Teachers perceived barriers in addressing racist incidents.**
    1.1. *Teachers' challenges*
        1.1.1. Estimating severity
        1.1.2. Determining severity
        1.1.3. Differentiation
        1.1.4. Difficult to intervene
        1.1.5. Effective approach
        1.1.6. Invisibility
        1.1.7. External influences
            1.1.7.1. Family
            1.1.7.2. Media
            1.1.7.3. Politics
    1.2. Teachers' perceived behavior of others
        1.2.1. Colleagues
        1.2.2. Families
        1.2.3. Parents
        1.2.4. Pupils
2. **The (un)intentionality and (un)awareness of pupils**
    2.1. Pupils (in general)
        2.1.1. Awareness
        2.1.2. Inner conflict
        2.1.3. Lack of knowledge
        2.1.4. Teaching culture
    2.2. Bully
        2.2.1. Unintentional
        2.2.2. Intentional

3. Racial identity

3.1. Awareness

3.2. (Lack of) experience

3.3. Personal interest

3.4. Relating to racism

4. Differentiation between racist and interpersonal bullying

4.1.1. No distinction

4.1.2. Sincerity of pupils

4.1.3. One time expressions

4.1.4. Comments

4.1.5. Having conversations

4.1.6. Intervening necessary

4.1.7. (in)visibility

4.1.8. Cause-effect deficiency

4.1.9. Creates unsafety victim

4.1.10. Everything you pay attention to grows

4.1.11. Unconscious behavior of pupils

4.1.12. Creates unsafe class climate

5. Racism and culture

5.1. Teachers' attitude towards racism

5.1.1. Cultural school dynamic

5.1.2. Multicultural society

5.1.3. Racism a recent problem

5.1.4. Teach about culture

5.1.4.1. Cultural diversity

5.1.4.2. Cultural awareness

5.1.4.3. Cultural differences

5.1.4.4. Cultural understanding

5.1.4.5. Positive cultural approach

5.1.5. Comfortable to discuss

5.1.6. Complex

5.1.7. Dare to discuss

5.1.8. Discuss if mentioned in curriculum

5.1.9. Link to current society

5.1.10. Open approach

5.1.11. Not discussed as stand-alone topic

5.2. Teachers' perceived competence in discussing racism

5.2.1. Capable

5.2.2. Comfortable

5.2.3. Depends on class climate

5.2.4. Accessibility of study material

5.2.5. Through conversations

5.2.6. Not difficult to discuss

5.2.7. Willingness to learn more

5.3. Teachers' attitude towards different cultures

5.3.1. Pupils' background

5.3.2. Create awareness

5.3.3. Reflection of current society

5.3.4. Reason to discuss

5.3.5. Not discussed in class

5.3.6. Respectful approach

5.4. Teacher training

5.4.1. Racism absent

5.4.2. No further training

6. Coping with racist incidents

6.1. Teachers' knowledge

6.1.1. Obviousness

6.1.2. Definition of racist bullying

6.1.3. Repetitiveness/long term

6.2. Victim

6.2.1. First attend to victim

6.2.2. Let victim feel understood

6.2.3. Let victim feel heard

6.2.4. Supportive

6.3. Bystanders

6.3.1. Acknowledge cultural strengths

6.3.2. Create cultural knowledge

6.3.3. Involvement of bystanders

6.3.4. Whole class approach

6.3.5. Depends on context

6.3.6. Depends on character

6.4. Bully

6.4.1. Address behavior

6.4.2. Create awareness

6.4.3. Change behavior

6.4.4. Change perspective

6.4.5. Approach parents

6.4.6. Disapproval

6.4.7. Punishment

6.4.8. Blind spot

6.5. Ineffective strategies racist incidents

6.5.1. General

6.5.1.1. Negative attention

6.5.1.2. Being too emotional

6.5.2. Victim

6.5.2.1. Degrading situation

6.5.2.2. Ignoring

6.5.2.3. Putting on the spot

6.5.3. Bystanders

6.5.3.1. Ignoring

6.5.3.2. Continuous involvement

6.5.3.3. Degrading situation

6.5.4. Bully

6.5.4.1. Anger

6.5.4.2. Directive response

6.5.4.3. Disapproval

6.5.4.4. Ignoring

6.5.4.5. No explanation

6.5.4.6. Punishment

6.5.4.7. Not being consistent

6.5.4.8. Putting on the spot

6.6. Effective strategies racist incidents

6.6.1.1. General

6.6.1.2. Engage conversation

6.6.1.3. Pro-activeness

6.6.2. Victim

6.6.2.1. Acknowledgement

6.6.2.2. Anonymity

6.6.2.3. Being approachable

6.6.2.4. Victim decides closure

6.6.3. Bystanders

6.6.3.1. Teach helping attitude

6.6.3.2. Involve other bystanders

6.6.4. Bully

6.6.4.1. Create understanding

6.6.4.2. Find cause

6.6.4.3. Involve parents

6.6.4.4. Listen

6.6.4.5. Punishment

6.6.4.6. Victimize bully

6.7. Teachers' perceived competence in addressing racist incidents

6.7.1. Engaging in conversation

6.7.2. Through experience

6.7.3. Willingness to learn more

6.8. Teachers' skills

6.8.1. Cultural knowledge

6.8.2. Setting boundaries

6.8.3. Communication

6.8.4. Listening

6.8.5. Openness

6.8.6. Persuasion

6.8.7. Consistency

6.8.8. Empathy

6.8.9. Fearlessness

6.8.10. Personal exposure

6.8.11. Sensibility

6.8.12. Being understanding

6.8.13. Respectful

6.8.14. Creating a safe class climate

7. Creating a safe class climate

7.1. Class climate

7.1.1. Creating safety

7.1.2. Cultural background pupils

7.1.3. External unsafe factors

7.1.4. Discussing racism

7.2. Group dynamic

7.2.1. Class hierarchy

7.2.2. Fear of bully

7.3. Student-teacher relationship

7.3.1. Trust

7.3.2. Individual relationships

7.3.3. Knowing the pupil

8. School climate, school materials and tools

8.1. School climate

8.1.1. Racism

8.1.1.1. Avoidance

8.1.1.2. Colleagues

8.1.1.3. Cultural awareness

8.1.1.4. Cultural excursions

8.1.1.5. Cultural holidays

8.1.1.6. Multicultural

8.1.1.7. Prioritize religion

8.1.1.8. Project religion

8.1.1.9. Not making it bigger than it is

8.1.1.10. Racism absent topic

8.1.2. Racist bullying

8.1.2.1. No distinction

8.1.2.2. Behavior expectations of pupils

8.1.2.3. Escalation ladder

8.1.2.4. No pro-active approach

8.1.2.5. Not included in policy

8.1.2.6. Teachers' responsibility

8.2. School materials and tools

8.2.1. As conversation starter

8.2.2. School methods

8.2.3. Usage depends on class climate

8.2.4. Limited material

8.2.5. No material

8.2.6. Racism present

8.2.7. Racism absent

8.2.8. To create awareness

8.2.9. Online

8.3. Teachers' needs

8.3.1. Background information

8.3.2. Case studies

8.3.3. Cross group material

8.3.4. Cross school material

8.3.5. Cultural experiences

8.3.6. Digital tools

8.3.7. Examples

8.3.8. Experiences

8.3.9. Excursions

8.3.10. Games

8.3.11. (Parental) Guide

8.3.12. Link to current society

8.3.13. Self-reflecting material

8.3.14. Stories

8.3.15. Week of racism
